# Gut microbiota modulation: a tool for the management of colorectal cancer

**DOI:** 10.1186/s12967-022-03378-8

**Published:** 2022-04-21

**Authors:** Yan Wang, Hui Li

**Affiliations:** Department of Gastroenterology, Shengjing Hospital of China Medical University, Shenyang, 110004 China

**Keywords:** Colorectal cancer, Gut microbiota, Probiotics, Fecal microbiota transplantation

## Abstract

Colorectal cancer (CRC) is the second cause of cancer death and the third most frequently diagnosed cancer. Besides the lifestyle, genetic and epigenetic alterations, and environmental factors, gut microbiota also plays a vital role in CRC development. The interruption of the commensal relationship between gut microbiota and the host could lead to an imbalance in the bacteria population, in which the pathogenic bacteria become the predominant population in the gut. Different therapeutic strategies have been developed to modify the gut immune system, prevent pathogen colonization, and alter the activity and composition of gut microbiota, such as prebiotics, probiotics, postbiotics, antibiotics, and fecal microbiota transplantation (FMT). Even though the employed strategies exhibit promising results, their translation into the clinic requires evaluating potential implications and risks, as well as assessment of their long-term effects. This study was set to review the gut microbiota imbalances and their relationship with CRC and their effects on CRC therapy, including chemotherapy and immunotherapy. More importantly, we reviewed the strategies that have been used to modulate gut microbiota, their impact on the treatment of CRC, and the challenges of each strategy.

## Background

According to GLOBOCAN estimate, colorectal cancer (CRC), with around 1.9 million new cases and 935,000 deaths, stands in the third and second ranks of incidence and mortality caused by cancer, respectively, in 2020 [1]. CRC can be regarded as a sign of socio-economic development. In countries undergoing significant transitions, the incidence tends to steadily increase with the increase in Human Development Index (HDI) [2, 3]. The increase in previously low-risk and low HDI countries may reflect changes in diet and lifestyle factors, such as the shift to a higher intake of animal-based foods and a more sedentary lifestyle, resulting in a reduction in physical activity and being overweight, which are associated with the risk of CRC [4]. Besides genetic, epigenetic, and environmental factors, the gut microbiome and its related parameters, including immunity, metabolism, and interaction with the host, determine the host’s health and disease [5].

Gut microbiota, the microorganisms that live in the gastrointestinal (GI) tract, not only include almost 3 × 10^13^ bacteria but also fungi, viruses, protists, and archaea [6]. Although the gut microbiota usually has a commensal relationship with its host, this relationship may be interrupted by changes in bacterial composition. The modulation occurs through an imbalance in the bacterial population, in which pathogenic bacteria replace harmless commensal bacteria. These pathogenic bacteria can invade the intestinal wall, cause inflammation, and induce carcinogenic signals and metabolites, leading to damage to the host [7]. Therefore, these bacteria may promote colon tumors; however, it is not clear whether these bacteria cause or the result of CRC [8]. With the increasing understanding of how the gut microbiota promotes cancer and affects the outcome of treatment, regulation of the gut microbiota aimed at restoring homeostasis of the gut microbiota has become a potential strategy for the prevention and treatment of CRC. This review aimed to summarize the role of the gut microbiota in CRC development and the different strategies applied to regulate and modulate gut microbiota.

## Gut microbiota in CRC

Although gut microbiota plays pivotal roles in gut homeostasis by various mechanisms, including acting as a defensive barrier against infection, developing the mucosal immune system, and involving in metabolic functions, there is growing evidence from large metagenomic studies that imbalance in human gut microbiota links with colorectal carcinogenesis [9]. In a study, Zhao et al*.* reviewed the cohort studies determining dysbiosis and the differences between the cancerous tissues and adjacent non-cancerous tissue of CRC patients and revealed the heterogeneity of microbiome in CRC [10]. Table 1 reviews some studies to describe the main differences between the phyla, genus, and species between healthy papulation and CRC patients.

**Table 1:** The differences between phyla, genus, and species between healthy papulation and CRC patients

Healthy sample			Tumor sample			
Phylum	Genus	Species	Phylum	Genus	Species	Refs.
Firmicutes Bacteriodetes Actinobacteria	*Faecalibacterium* *Prevotella*	*B. vulgatus* *F. prausnitzii*	Fusobacteria Proteobacteria Spirochaetes	*Fusobacterium* *Treponema*	*B. fragilis* *F. nucleatum*	[11]
Firmicutes	*Agathobacter* *Anaerostipes* *Butyricicoccus A* *Butyrivibrio A* *CAG-41* *Eubacterium G* *Eubacterium R* *Faecalibacterium* *Lachnospira* *GCA-900066135* *TF01-11* *UBA11524*	*A. hadrus* *B. catenulatum* *F. saccharivorans* *F. prausnitzii* *A. rectalis* *A. faecis*	Bacteroidota Desulfobacterota Fusobacteriota	*Anaerotignum* *Bilophila* *Bulleidia* *Flavonifractor* *Gemella* *Intestinimonas* *Parvimonas* *Peptostreptococcus* *Porphyromonas* *Prevotella* *Ruthenibacterium*	*E. coli D* *P. distasonis* *B. fragilis* *A. finegoldii* *A. onderdonkii* *A. muciniphila* *B. thetaiotaomicron* *M. torques* *R. gnavus* *Porphyromonas* species	[12]
Bacteriodetes Firmicutes Actinobacteria	*Faecalibacterium* *Prevotella*	NA	Proteobacteria Fusobacteria	*Bacteroides* *Escherichia* *Sutterella*	NA	[13]
Firmicutes Actinobacteria Bacteriodetes	*Bacteroides* *Blautia* *Faecalibacterium*	*F. prausnitzii* *B. faecis* *S. termitidis* *A. shahii* *B. uniformis*	Fusobacteriota Proteobacteria	*Fusobacterium* *Prevotella* *Parvimonas*	*F. nucleatum* *C. Koseri* *T. socranskii* *L. trevisanii*	[14]
Firmicutes Actinobacteria	*Bacillus* *Lactococcus* *Acinetobacter* *Pseudomonas* *Parabacteroides*	NA	Bacteriodetes Proteobacteria Fusobacteria	*Fusobacterium* *Prevotella* *Alloprevotella* *Porphyromonas* *Peptostreptococcus* *Parvimonas*	NA	[15]
Firmicutes Euryarchaeota Spirochaetes	*Prevotella* *Faecalibacterium* *Lactobacillus* *Parabacteroides*	NA	Fusobacteria Proteobacteria Bacteriodetes	*Bacteroides* *Fusobacterium* *Bifidobacterium* *Streptococcus* *Halomonas* *Sphingomonas*	NA	[16]

**Fig. 1:**
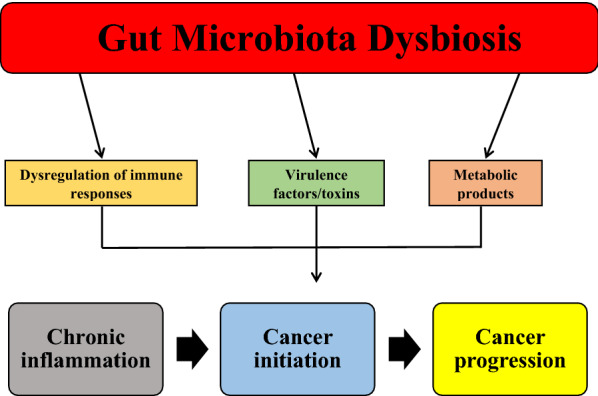
Gut microbiota dysbiosis and its relationship with CRC. Dysbiosis of gut microbiota and increasing the abundance of pathogenic microbiota could promote chronic inflammation and, subsequently, cancer initiation and progression through three mechanisms, including dysregulation of immune responses, virulence factors/toxins, and metabolic products

There is an abundance of *Helicobacter pylori*, *Fusobacterium nucleatum*, *Escherichia coli*, *Bacteroides fragilis*, *Peptostreptococcus anaerobius*, *Helicobacter hepaticus*, *Porphyromonas gingivalis*, *Enterococcus faecalis*, and *Streptococcus gallolyticus* has been related to CRC development [17]. Table 2 summarizes the abundance of some bacteria in patients with CRC. Certain species can develop CRC through specific and a variety of mechanisms. Mainly, three mechanisms have been identified for tumor-promoting activities of microbiota: pathogenic bacterial virulence factors/toxins, bacterial metabolic products, and immune modulation/reaction [18]. For example, colibactin or typhoid toxin secreted by *E. coli* or *Salmonella*, respectively, produces pro-inflammatory cytokines and bacterial adherence. Also, *B. fragilis* and *F. nucleatum* provide a favorable microenvironment for inhibitory immune cells [19]. Figure 1 indicates the main pathogenic mechanisms in CRC that involve the gut microbiome.

**Table 2:** Various bacteria abundance in patients with CRC

Bacteria	Detection method	Sample (n)	Positive percentage	Sample type	Affiliation	Refs.
*H. pylori*	Multiplex serology	4063	41%	Serum	USA	[20]
*H. pylori*	ELISA	1712	46.1%	Serum	Germany	[21]
*H. pylori*	Multiplex serology	1488	90%	Serum	Spain	[22]
*F. nucleatum*	qPCR	160	66.9%	FFPE tissue	Korea	[23]
*F. nucleatum*	qPCR	100	75%	Frozen tissue	Japan	[24]
*F. nucleatum*	qPCR	39	82.1%	Tissue	Korea	[25]
*E. coli*	PCR	31	90%	Tissue	Germany	[26]
*E. coli*	PCR	48	16.7%	Tissue	Malaysia	[27]
*E. coli*	PCR	48	83%	Tissue	Iran	[28]
*B. fragilis*	PCR	49	88.5%	Mucosal tissue	USA	[29]
*B. fragilis*	PCpR	60	58.3%	Stool	Iran	[30]
*P. anaerobius*	qPCR	154	NA	Mucosa/Stool	China	[31]
*P. gingivalis*	qPCR	31	32.2%	Tissue	China	[32]
*P. gingivalis*	PCR	71	76%	Saliva	Turkey	[33]
*E. faecalis*	PCR	20	NA	Stool	India	[34]
*E. faecalis*	qPCR	9	22.2%	Stool	Italy	[35]
*S. gallolyticus*	qPCR	148	74%	Tissue	USA	[36]
*S. gallolyticus*	qPCR	190	3.2%	Tissue	Spain	[37]

*ELISA* enzyme-linked immunosorbent assay, *qPCR* quantitative real‐time polymerase chain reaction, *FFPE* formalin‐fixed paraffin‐embedded

### Helicobacter pylori

It has been shown that infection with *H. pylori* is associated with an increased risk of CRC. For instance, Teimoorian et al*.* found that *H. pylori* infection was considerably more prevalent in patients with adenomatous polyps and colon cancer than in healthy subjects [38]. A meta-analysis study, including 47 studies with 17,416 CRC cases and 55,811 control cases, revealed a positive association between infection with *H. pylori* and increased risk of CRC, although this association was related to the region of the study [39]. As virulence factors in some *H. pylori*, cytotoxin-associated gene A (CagA) and vacuolating cytotoxin A (VacA), can promote and activate inflammation pathways [40]. Furthermore, there is evidence that direct and indirect production of reactive oxygen species (ROS) and reactive nitrogen species (RNS) by some strains of *H. pylori* participate in colon tumorigenesis [41]. There are also other hypotheses about the indirect contribution of *H. pylori* to CRC carcinogenesis, including changes in the colonization of the intestine with other bacteria and an increase in the gastrin release [42].

### Fusobacterium nucleatum

In a meta-analysis study, Gethings-Behncke et al*.* found a higher abundance of *F. nucleatum* in CRC patients' tissue and fecal samples. They also indicated that the high *F. nucleatum* abundance in CRC patients was associated with poorer overall survival [43]. Two other meta-analysis studies confirmed that intestinal and fecal *F. nucleatum* could be a valuable diagnostic marker for CRC [44, 45]. Various mechanisms are involved in CRC tumorigenesis of *F. nucleatum*, including virulence factors, metabolism products, immune modulation, and miRNAs [46]. For instance, the Fap2 protein of bacteria mediates *F. nucleatum* enrichment during CRC by binding to tumor-expressed D‐galactose‐β(1–3)‐N‐acetyl‐D‐galactosamine (Gal‐GalNAc) [47]. FadA is another virulence factor of *F. nucleatum* that activates the β‐catenin pathway by binding to E‐cadherin, leading to an increase in the levels of Wnt7b, NFκB, cyclin D1, and Myc [48]. Moreover, *F. nucleatum* enhances angiogenesis by increasing the expression of CD31 and CD34 as adhesion molecules and vascular endothelial growth factor receptors 1 and 2 (VEGFR1 and VEGFR2) [49]. Angiogenesis is a hallmark of cancer that provides oxygen and nutrients for tumor cells to guarantee their growth [50, 51].

### Escherichia coli

There is accumulating evidence that *E. coli* are frequently colonizing in CRC lesions and adjacent epithelium [52, 53]. For instance, Iyadorai et al*.* found that *pks* + *E. coli* is more frequent in patients with CRC than in healthy cases and the *pks* + *E. coli* strains play an important role in the initiation and development of tumors [27]. It has been shown that *E. coli* could induce CRC in IL-10-deficient mice, proposing that inflammation is pivotal for tumorigenesis [54]. In addition, pathogenic *E. coli* can synthesize toxins called cyclomodulins, including cycle inhibiting factor, cytotoxic necrotizing factor (CNF), colibactin, and cytolethal distending toxins (CDT), which are genotoxin or interfere with the cell cycle [55, 56]. *E. coli* also promotes the survival of tumor cells by inducing macrophage inhibitory cytokine 1 (MIC-1), leading to an increase in the expression of transforming growth factor β-activated kinase 1 (TAK1) and subsequently RhoA GTPase after pathogen infection, and sustained COX-2 expression [57, 58].

### Bacteroides fragilis

The *B. fragilis* is categorized into two subtypes, including enterotoxigenic *B. fragilis* (ETBF) and nontoxigenic *B. fragilis* (NTBF) [59]. It has been reported that ETBF was considerably more abundant in precancerous and cancerous lesions of CRC than in healthy controls and mucosal *B. fragilis* toxin (BFT) could be considered a risk factor and screening marker for CRC development [60]. BFT is a zinc-dependent metalloprotease toxin involved in the transduction of epithelial cell signals in the colon, such as Wnt, NFκB, and MAPK pathways. It induces inflammatory mediators' production and facilitates CRC development [61]. Also, ETBF could induce colitis and tumor formation in an IL-17-dependent manner in Min^Apc±^mice through STAT3 activation [62]. The activation of these pathways results in the recruitment of immature myeloid cells by inducing CXC chemokines, leading to the creation of a pro-inflammatory environment [63].

### Peptostreptococcus anaerobius

Metagenomic analysis of mucosal and stool samples from patients with CRC discovered that *P. anaerobius* is another gut bacteria enriched in CRC [64, 65]. Tsoi et al*.* reported that mice exposed to *P. anaerobius* showed a higher incidence of colon dysplasia than those not given the bacteria (63% versus 8.3%), and colon cells exhibited considerably higher levels of proliferation when exposed to *P. anaerobius* compared with control cells. Mechanistically, *P. anaerobius* promotes tumorigenesis by activating toll-like receptors 2 and 4 (TLR2 and TLR4) to enhance the intracellular levels of ROS, which increase cholesterol synthesis and subsequently cell proliferation [31]. In another study, Long et al*.* showed that *P. anaerobius* preferentially attaches to CRC cell lines compared to normal intestinal epithelial cells. They identified putative cell wall binding repeat 2 (PCWBR2), a surface protein of *P. anaerobius*, responsible for this adherence. The PCWBR2 directly binds to integrin α2/β1, a frequently overexpressed receptor in CRC tumors, which promotes the activation of the PI3K-Akt signaling pathway in CRC cells, resulting in activation of NFκB and increase of cell proliferation. The activation of NFκB increases pro-inflammatory responses and the levels of IL-10 and IFN-γ *P. anaerobius*-treated mice. Furthermore, *P. anaerobius*-treated mice exhibited a significant increase in the population of tumor-associated macrophages (TAMs), granulocytic tumor-associated neutrophils, and myeloid-derived suppressor cells, which are involved in chronic inflammation and tumor development [66].

### Porphyromonas gingivalis

*P. gingivalis* is an oral microbiota that can be transmitted to the large intestine and contribute to the pathogenesis of various diseases [67]. It has been shown that oral administration of *Prevotella intermedia* and *P. gingivalis* in mice led to systemic inflammation, endotoxemia, disruption of the intestinal barrier, and intestinal dysbiosis [68]. Recently, Mu et al*.* investigated the role of *P. gingivalis* in CRC and its mechanism of action. They found that *P. gingivalis* could adhere and invade CRC cells, significantly enhance their proliferation, and increase the percentage of cells in the S-phase of the cell cycle. Mechanistically, *P. gingivalis* contributes to the proliferation of CRC cells by significantly activating the MAPK/ERK pathway [69]. In another study, Wang et al*.* demonstrated that oral gavage of *P. gingivalis* to Apc^Min/+^ mice promotes CRC tumorigenesis and modulates tumor microenvironment (TME) by preferentially increasing myeloid-derived immune cells, such as macrophages and DCs, and inducing a pro-inflammatory condition. They also indicated that *P. gingivalis* promotes CRC by activating nucleotide-binding oligomerization domain-like receptor family pyrin domain-containing 3 (NLRP3) inflammasome [32].

### Enterococcus faecalis

There are controversial data about the role of *E. faecalis* in CRC: some studies exhibited no role or a protective role against CRC while others showed a harmful role [70]. For instance, De Almeida et al*.* found a decreased abundance of *E. faecalis* in the feces of CRC patients compared to healthy cases (22.2% versus 44.4%) [35]. Similarly, Viljoen et al*.* did not find a considerable clinical association between infection with *E. faecalis* and CRC [71]. On the other hand, Wang et al*.* indicated that *E. faecalis* contributes to inducing CRC via activating the Wnt/β-catenin pathway and inducing pluripotent transcription factors. They showed that the exposure of colonic epithelial cells to *E. faecalis*-infected macrophages promotes the initiation of CRC through chromosomal instability, gene mutation, and cell transformation [72].

### Streptococcus gallolyticus

It has been shown that colonization of *S. gallolyticus*, previously called *S. bovis*, is significantly correlated with CRC development [73, 74]. A case–control study on 109 cases revealed that *S. gallolyticus* was significantly higher among colorectal neoplasia patients than in control cases (70% versus 32%) [75]. Mechanistically, *S. gallolyticus* antigen could promote cell proliferation and angiogenesis by inducing COX-2 expression [76]. In addition, the wall-extracted antigen of *S. gallolyticus* induces the production of inflammatory cytokines by binding to CRC cell lines [77].

### Gut microbiota and chemotherapy implications

There is emerging evidence that host response to chemotherapy can be modulated with gut microbiota through various mechanisms, including immunomodulation, microbial translocation, educed ecological diversity, enzymatic degradation, and metabolism [78].

Viaud et al*.* investigated the effect of cyclophosphamide (CTX) and doxorubicin on the composition of small intestine microbiota in mouse models. They found that treatment with both chemotherapy agents led to shortening the small intestine's villi, discontinuity in the intestinal epithelial barrier, accumulation of inflammatory cells, and translocation of commensal bacteria into secondary lymphoid organs. Although treatment with CTX did not reduce the total bacterial counts in mice's small intestine after seven days, the abundance of enterococci and lactobacilli showed a reduction. In addition, some Gram-positive bacteria, such as *Enterococcus hirae*, *Lactobacillus murinus*, and *Lactobacillus johnsonii*, stimulated the generation of pathogenic” T helper 17 (pTh17) and type 1 T helper (Th1) cells, whereas killing these bacteria with antibiotics reduced pTh17 responses and enhanced tumor resistance to CTX [79]. In another study, Daillère et al*.* demonstrated that *Barnesiella intestinihominis* and *Enterococcus hirae* are involved in the host response to CTX. They found that *B. intestinihominis* accumulates in the colon and induces intratumoral re-instating of IFN-γ-producing γδT cells, whereas *E. hirae* translocates to secondary lymphoid organs from the small intestine and increases the ratio of CD8/Treg in the TME. The colonic *B. intestinihominis* acts as an orchestrator of CTX effects and *E. hirae* could restore CTX-induced anti-tumor effects [80]. Yu et al*.* demonstrated that *F. nucleatum* could promote resistance of CRC cells to 5-fluorouracil (5-FU) and oxaliplatin by activating autophagy. From a mechanistic view, *F. nucleatum* involved TLR4 and MYD88, downregulating the expression of miR-4802 and miR-18a*. The reduction in miR-4802 and miR-18a* levels leads to the upregulation of ATG7 and ULK1, respectively, resulting in the activation of autophagy [81]. Another example of gut microbiota and modulation of chemotherapy is the control of side effects and metabolism of irinotecan, a pro-drug that is activated in the form of SN-38, a topoisomerase I inhibitor. The host liver enzymes could glucuronide SN-38 and convert it to an inactivate form (SN-38G). After reaching the intestine, SN-38G is hydrolyzed back to SN-38 through bacterial β-glucuronidase enzymes, which leads to severe diarrhea and intestinal damage [82].

### Gut microbiota and immunotherapy implications

The cancer immunotherapy approach has become a promising therapeutic way to treat cancer in which the immune responses of the patient are elicited to exert anti-tumor effects. There is evidence that gut microbiota could modulate response to immunotherapy. It has been shown that specific gut bacteria could elevate tumor response in immunotherapy, including *Eubacterium limosum*, *Alistipes shahii*, *B. fragilis*, *Akkermansia muciniphila*, and *Faecalibacterium spp* [83–87]. For instance, the results of a study revealed that a significant increase in the abundance of *Bifidobacterium* could elevate the levels of tumor-infiltrating lymphocytes (TILs) and facilitate tumor response to treatment with programmed cell death protein 1 ligand 1 (PD-L1) antibody [88]. Recently, Shi et al*.* indicated that gut microbiota could affect the efficacy of anti-CD47 immunotherapy by changing TME and gut immunity. *Bifidobacterium* could accumulate within TME and convert non-responder tumors to responder ones to immunotherapy with anti-CD47 in an IFN- and stimulator of interferon genes (STING)-dependent manner [89]. In another study, Lv et al*.* found that the efficacy of PD-1 blockade could be enhanced through gut microbiota remodeling in CRC with microsatellite stability. They found that a traditional Chinese drug, Gegen Qinlian decoction (GQD), could enhance the efficacy of immunotherapy with anti-PD-1 antibody and inhibit tumor growth in vivo. The combination of GQD and anti-PD-1 modulates gut microbiota, increasing *Bacteroides acidifaciens* and *Bacteroidales S24-7* family. The combinational therapy also remarkably increased CD8 + T cells proportion in tumor tissues and peripheral blood and elevated the expression of IFN-γ and IL-2. Mechanistically, the combination of GQD and anti-PD-1 increased the metabolism of glycerophospholipid and sphingolipid pathways [90]. It has been shown that both metabolites could act as biomarkers in monitoring CRC patients [91, 92].

## Strategies of gut microbiota modulation

There is evidence that gut microbiota and microbial-secreted metabolites could be targeted as a therapeutic strategy in combating CRC. For instance, Bhalla et al*.* showed that microbial metabolites, such as folate, could suppress CRC cells' viability; thus, modulation of gut microbiota to produce anti-cancer metabolites could be used to treat CRC [93]. The following section will consider the strategies that have been applied to modulate gut microbiota as a therapeutic strategy.

### Probiotics

**Fig. 2:**
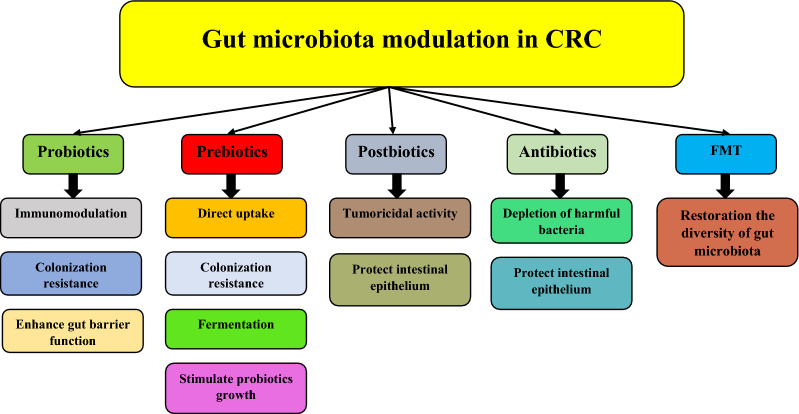
Strategies applied for modulating gut microbiota in CRC and the mechanisms of action of each strategy. Probiotics: Probiotics exert their effects on gut microbiota composition through immunomodulation, inhibition of pathogenic bacteria colonization, and enhancement of the gut barrier functions. Prebiotics: They act as gut microbiota modulatory elements through direct uptake by the intestine and exerting anti-inflammatory activities, prevention of the colonization of pathogens by interacting with them, fermentation by intestinal microbiota, and stimulation of beneficial gut bacteria. Postbiotics: They exert their tumoricidal functions through selective inhibiting tumor cells and protecting intestinal epithelium by inhibiting apoptosis in epithelial cells and increasing IgA secretion. Antibiotics: Antibiotics could deplete the intestine from harmful bacteria and preserve intestinal epithelium. FMT: This strategy helps restore the diversity of microbiota in the gut

Probiotics are microorganisms, including bacteria, yeasts, and molds, that can improve the host health when delivered in an adequate quantity. *Lactobacillus*, *Bacillus*, *Bifidobacterium*, *Streptococcus*, and *Enterococcus* are the most used bacterial genera as probiotics [94]. Probiotics affect the gut in the prevention and treatment of CRC through three main mechanisms: 1) immunomodulation, 2) inhibition of pathogenic bacteria colonization, and 3) enhancement of the gut barrier functions. Figure 2 represents the strategies applied for gut microbiota modulation and the mechanisms of action of each strategy.

Dysbiosis conditions could activate pathways and transcriptional factors, such as MAPK and NF-κB, that increase the production of nitric oxide (NO) and IL-8 as pro-inflammatory cytokines, leading to the occurrence of inflammatory bowel disease (IBD) and CRC. There is accumulating evidence that probiotics administration re-establishes the balance of gut microbiota by increasing the secretion of anti-inflammatory cytokines, such as TGF-β2 and IL-10, through regulatory T (Treg) cells [95]. For example, *Bifidobacterium breve* and *Bifidobacterium infantis* could activate colonic dendritic cells (DC), resulting in the expression of Foxp3^+^ Treg and IL-10 producing type 1 Treg (Tr1) [96, 97]. Lee et al*.* demonstrated that *Bacillus polyfermenticus* containing probiotics properties exhibited an anti-proliferative effect on CRC cell lines, HT-29 and LoVo, by reducing the levels of pro-inflammatory cytokines, including TNF-α, NO, and COX-2 [98]. On the other hand, it has been shown that probiotics bacteria could induce pro-inflammatory responses. Hradicka et al*.* studied the immunomodulatory effects of six lactobacilli containing probiotic properties in an in vitro model of macrophage cells and their anti-tumor properties in a rat model of CRC. They indicated that the bacteria could induce the release of pro- and anti-inflammatory cytokines, including IL-1β, TNF-α, IL-18, and IL-23, in co-culture of lactobacilli with M1- and M2-like macrophages, whereas the bacteria oral administration led to a decrease in tumor multiplicity, numbers, and volume, as well as restore of colon length and increase in the production of IL-18 [99]. In another study, a clinical trial found that oral administration of probiotic *Saccharomyces boulardii* could downregulate the levels of both pro- and anti-inflammatory cytokines, including IL-10, IL-23A, and IL-1β, while the incidence of infectious complications in patients receiving probiotic was 13.3% compared to 38.8% in the control group [100].

The other beneficial property of probiotics is the inhibition of colonization of pathogenic bacteria that degrade the gut and release toxic compounds. Probiotics could inhibit the proliferation of detrimental microorganisms by lowering luminal pH, producing anti-microbial peptides, and reducing pro-tumorigenic enzymes [101]. For instance, Fayol-Messaoudi et al*.* demonstrated that probiotic strains of *Lactobacillus* could inhibit the growth of the pathogenic bacteria, *Salmonella enterica*, by producing non-lactic acid and lactic acid substances and subsequently lowering pH [102]. In addition, there is evidence that probiotics could prevent colon infection with pathogenic bacteria, including *Staphylococcus aureus* and *Clostridium difficile* [103, 104]. Probiotics also could inhibit the adhesion of pathogens to the intestine. Collado et al*.* found that probiotic strains can displace adhered pathogens or inhibit the adhesion of pathogens to intestinal mucus, while different combinations of probiotics showed an enhanced percentage of adherence inhibition [105]. In another study, Behbahani et al*.* revealed the adhesion ability of the probiotic *L. plantarum* strain L15 to the intestinal cells. This strain exhibited anti-adherence activities, such as competition, inhibition, and replacement features, against *E.coli* [106].

Intestinal epithelium acts as a physical barrier and protects the lamina environment from pathogenic toxins, stress factors, and bacteria. The barrier function ha**s** three components: tight junction (TJ), adhesion protein junction (AJ), and desmosomes. It has been shown that disruption of this physical barrier results in the development of IBD and IBD-associated CRC, as well as an acceleration in tumor invasion and metastasis [107, 108]. There is evidence that some probiotic strains improve the function of the gut barrier. A clinical trial study revealed that oral administration of probiotics both pre-operatively and post-operatively in CRC patients undergoing surgery could increase transepithelial resistance and upregulate the expression of TJ. Patients receiving probiotics had increased bacterial variety in fecal and decreased enteropathogenic bacteria in the blood [109]. Liu et al*.* conducted a meta-analysis study to investigate the effect of probiotics on the gut barrier in CRC patients after operation. Assessment of 17 studies demonstrated that probiotics administration could protect the physical and biological barrier in the intestine by increasing intestinal permeability, occludin levels, secretory immunoglobulin A (SIgA) levels, and decreasing bacterial translocation [110]. In another study, the administration of probiotic strains, including *L. acidophilus*, *B. bifidum*, and *B. infantum* (LBB regimen), could inhibit CRC development by reducing tumor count, tumor size, and tumor incidence. LBB treatment also altered the gut microbiota and decreased the abundance of pathogenic bacteria. Furthermore, the LBB regimen enhanced the integrity of the intestinal epithelial and mucosal barrier by increasing the expression of occludin, zonula occludens-1 (ZO-1), and mucin 2 (MUC2). LBB treatment also prevented CRC via enhancing TLR2 expression and downregulation of TLR4, COX2, caspase-3, and β-catenin. These results suggested that the anti-cancer effects of probiotic LBB treatment was due to TLR2-dependent intestinal barrier enhancement and inhibition of inflammation, apoptosis, and β-catenin pathway [111]. Table 3 summarizes the application of probiotics in the management of CRC patients.

**Table 3:** Probiotics administration for the management of CRC patients

# of patients	Intervention	Duration	Outcome	Refs.
27	6 viable microorganisms of *Lactobacillus* and *bifidobacteria*	Twice daily 107 mg (orally) probiotics for 6 months	Reduced the level of pro-inflammatory cytokines, including TNF-α, IL-6, IL-10, IL-12, IL-17A, IL-17C and IL-22	[112]
57	2 mg *Enterococcus faecalis* T110, 10 mg *Clostridium butyricum* TO-A, and 10 mg *Bacillus mesentericus* TO-A	Daily (six tablets/day) for 15 days	Reduced the incidence of post-operative complications, including superficial incisional infections, time of meal intake, and time of flatus	[113]
8	1.4 × 10^10^ CFUs *Bifidobacterium lactis* Bl-04, 7 × 10^9^ CFUs *Lactobacillus acidophilus* NCFM, and 0.63 g inulin	Daily (two tablets/day) 8–78 days	- Increased bacterial diversity - Increased the abundance of butyrate-producing bacteria, including *Faecalibacterium* and *Clostridiales spp* - Reduced CRC-associated genera, including *Fusobacterium* and *Peptostreptococcus*	[114]
84	*Lactobacillus acidophilus* LA-5 (1.75 × 10^9^ cfu), *Lactobacillus plantarum* (0.5 × 10^9^ cfu), *Bifidobacterium lactis* BB-12 (1.75 × 10^9^ cfu), and *Saccharomyces boulardii* (1.5 × 10^9^ cfu)	One day before operation and continuing for another 15 days post-operatively	- Decreased the rate of all postoperative major complications, including pneumonia, anastomotic leakage, and surgical site infections - Shortened the time until hospital discharge	[115]
98	*Lactobacillus rhamnosus* GG supplementation (twice daily at a dose of 1–2 × 10^10^) and fiber (11 g guar gum per day) during chemotherapy	For 24 weeks	Reduced the frequency of diarrhea and abdominal discomfort	[116]
70	6 viable microorganisms of *Lactobacillus* and *bifidobacteria* plus omega-3 fatty acid at a dose of 2 g	- Probiotics for 4 weeks - Omega-3 fatty acid for 8 weeks	- Improved the overall quality of life - Alleviated certain side effects of chemotherapy - Reduced inflammatory biomarkers, including IL-6	[117]
28	2 × 10^9^ cfu *Lactobacillus rhamnosus* R0011 and *Lactobacillus acidophilus* R0052	For 12 weeks	- Decreased irritable bowel symptoms - Improved cancer-related quality of life	[118]

### Prebiotics

Prebiotics are “selectively fermented ingredients that cause specific changes in the gastrointestinal microbiota's composition and/or activities that confers benefits upon host well-being and health” [119]. The polyphenols, polyunsaturated fatty acids (PUFAs), and carbohydrates, including galactooligosaccharides (GOS), xylooligosaccharides (XOS), fructooligosaccharides (FOS), fructans, and inulin, possess prebiotic properties. Prebiotics exert their functions through various mechanisms: (1) stimulation of beneficial gut bacteria, (2) fermentation through intestinal microbiota, (3) direct uptake by the intestine and exerting anti-inflammatory activities, and (4) prevention of the colonization of pathogens by interacting with them [5].

It has been shown that prebiotic administration could enhance the abundance of probiotics, including *Akkermansia*, *Rosebura, Ruminococcus*, *and Faecalibacterium* [120, 121]. Zheng et al*.* prepared prebiotics-encapsulated probiotic spores (spores-dex) in which *C. butyricum* (as a probiotic) and chemically modified dextran (as a prebiotic) were combined, and their anti-cancer efficacy was assessed in colon tumor models. They demonstrated the enrichment of colon cancers with spores-dex following oral administration. The fermentation of dextran in the lesions by *C. butyricum* led to the production of short-chain fatty acids (SCFAs) with anti-cancer activities. Furthermore, spores-dex could increase SCFA-producing bacteria, including *Roseburia* and *Eubacterium*, and significantly inhibit tumor growth. SCFA-producing bacteria contribute to tumor inhibition by building a tumor-suppressing microenvironment in the intestine [122]. As SCFAs, butyrate is the main energy fuel for colonocytes, while acetate and propionate are metabolized by muscle and liver for energy generation and gluconeogenesis [123]. Moreover, butyrate could induce CRC apoptosis, modulate oxidative stress, enhance epithelial barrier, and downregulate inflammation [124]. In addition to their histone deacetylase inhibitory and intracellular metabolism activities, SCFAs exert most of their functions through G-protein coupled receptors (GPCRs) in the intestine, named GPR43 (FFA2), GPR41 (FFA3), and GPR109A. For instance, the binding of butyrate, acetate, and propionate to FFA2 in the colon epithelium triggers signaling cascades, which leads to cell cycle arrest, promoting apoptosis, and inhibition of inflammation. The expression of FFA2 enhances the growth of the *Bifidobacterium family* and inhibits the *Prevotellaceae* family and *H. hepaticus*. On the other hand, the butytae-producing Butyricicoccus pullicaecorum exhibits anti-tumorigenesis function by increasing the expression of FFA2 [125].

Prebiotics also interfere with pathogenic bacteria's adhesion to the epithelial cells and the intestine. For instance, Ribeiro et al*.* showed that olive pomace powders with prebiotic properties not only promote the production of SCFAs by microbiota but also exhibit anti-oxidant anti-adhesive activities against pathogens. They demonstrated that pulp-enriched powder, as the primary source of insoluble dietary fiber, inhibited the adhesion of *B. cereus* and *L. monocytogenes* up to 22% and 20%, respectively [126]. In another study, Leong et al. investigated the prebiotic properties of oligosaccharides in goats’ milk-based infant formulas and their mechanisms of action. They reported that the prebiotic oligosaccharides could remarkably enhance the growth of *lactobacilli* and *bifidobacteria* and reduce *S. typhimurium* and *E. coli* NCTC 10,418 adhesion to CRC cell line Caco-2 [127]. Because of structural similarities between the oligosaccharides in goats’ milk and carbohydrates on the gut surface, these oligosaccharides, especially fucosylated and sialylated ones, reduce the adhesion of pathogenic bacteria to intestinal epithelial cells via acting as soluble analogs for host cell receptors or changing such structures` expression [128, 129].

### Postbiotics

Postbiotics are defined as cell fractions, inactivated microbial cells, and cell metabolites made with probiotic live cells during fermentation and contained various health benefits for the host. Since the postbiotics are present in the conditioned/supernatants medium of bacterial culture, they are safer than viable microorganisms. Postbiotics exert their anti-tumor activities by: 1) selectively inhibiting tumor cells and 2) protecting intestinal epithelium through the inhibition of apoptosis in epithelial cells and increasing IgA secretion [5].

It has been shown that postbiotic metabolites produced by some bacteria, such as *L. plantarum*, have cytotoxic and anti-proliferative effects on tumor cells, including CRC cells [130, 131]. Lee et al*.* assessed the tumoricidal function of probiotic cell-free supernatant treatment using *L. fermentum* against CRC cells in a 3D culture system. They found that the bacterial culture supernatant could induce apoptosis of CRC cell lines by upregulating Bax, Bak, Bid, Noxa, and caspase-3 [132]. In another study, Konishi et al*.* demonstrated the tumor-suppressive effect of ferrichrome in the culture supernatant of *L. casei* ATCC334 against colon cancer cells. Ferrichrome reduced the cancer cell viability by inducing c-jun N-terminal kinase (JNK)-mediated apoptosis. Furthermore, the tumoricidal effect of ferrichrome on cancer cells was greater than 5-FU and cisplatin, whereas the toxicity of ferrichrome against non-cancerous intestinal cells was less than the chemotherapy agents [133].

Postbiotics' other mechanism of action is associated with their ability to suppress intestinal inflammation and maintain the gut barrier's integrity. For instance, Izuddin et al*.* showed that supplementation of post-weaning lambs with postbiotic derived from *L. plantarum* RG14 led to ruminal epithelial growth, downregulation of pro-inflammatory cytokines, including TNF and IL-1β, and anti-inflammatory cytokine IL-10, and improvement of intestinal barrier integrity via upregulating tight junction protein 1 (TJP1), claudin-1 (CLDN-1), and CLDN-4 [134]. In another study, Gao et al*.* identified HM0539, a secreted protein, beneficial effects in the culture of *L. rhamnosus* GG. They found that HM0539 plays a protective role in maintaining the integrity of the intestinal barrier by increasing the expression of intestinal mucin and preventing intestinal barrier injury [135]. Another postbiotic protein of *L. rhamnosus* GG that affects the intestinal epithelium is p40 protein. To protect it from degradation, Yan and Polk used hydrogel-coating p40 and indicated that p40 protein promotes a protective immune response, reduces apoptosis of intestinal epithelial cells, and protects barrier function of the colon in an epithelial growth factor (EGF) receptor-dependent manner [136].

### Antibiotics

It has been shown that the use of antibiotics has negative impacts on gut microbiota, such as reduction in the biodiversity of bacteria, selection of antibiotic-resistant organisms, and changes in metabolic functions, resulting in antibiotic-associated diarrhea as well as recurrence of *C. difficile* infection [137]. Although there is increasing evidence that antibiotics increase CRC risk [120, 121], they could also decrease the size and number of tumors by manipulating the gut microbiome [138, 139]. For example, DeStefano Shields et al*.* reported that treatment with cefoxitin antibiotic could completely and durably clear enterotoxigenic *B. fragilis* colonization in an intestinal neoplasia mice model [140].

Antibiotics also play a protective role in maintaining the mucosal barrier in the intestine. Since high consumption of red meat increases the risk of CRC, Ijssennagger et al*.* investigated the effect of heme, as a pigment and proxy of red meat, on gut microbiota and CRC development. They demonstrated that mice fed a diet containing heme exhibited damaged intestinal epithelium and hyperproliferation, leading to colon cancer, whereas heme + antibiotic regimen-received mice did not show epithelium damage and hyperproliferation. Mechanistically, hydrogen sulfide produced by microbial exposes the intestinal epithelium to cytotoxic heme via opening the mucus barrier. Antibiotics inhibit the production of microbial sulfide, thereby maintaining the integrity of the mucus barrier that prevents the induced hyperproliferation [141]. Despite the mentioned mechanisms, the relationship between gut microbiota, antibiotics, and CRC is very complicated and should be studied carefully to determine this relationship and its application in CRC therapy.

### Fecal microbiota transplantation

Fecal microbiota transplantation (FMT), an interested and most innovative biotherapeutic method, is defined as transferring stool transplants from healthy individuals into patients. Although FMT is the approved method for treating *C. difficile* infection (CDI) [142], it also showed promising potential for treating obesity, IBD, diabetes, non-alcoholic fatty liver disease, and cardiovascular diseases. A prospective clinical trial study revealed that FMT could inhibit intestinal colonization of antibiotic-resistant bacteria (ARB) in patients with blood disorders. FMT led to completely (75%) and partially (80%) decolonization of ARB from patients [143]. FMT tries to restore gut microbiota diversity. It has been shown that FMT could restore microbial homeostasis by introducing a disease-free and healthy microbial population to an unbalanced community and act as a useful tool for ameliorating several GI disorders, including CDI, IBD, and irritable bowel syndrome [144]. Rosshart et al*.* showed that wild mice fecal transplantation to laboratory mice could promote fitness and improve resistance to colorectal tumorigenesis induction through mutagen/inflammation agents [145]. Interestingly, Wong et al*.* demonstrated that fecal microbiota from CRC patients increased tumor formation and reduced microbial abundance in conventional and germ-free mice given azoxymethane, as a carcinogen. The mice also increased the proportion of Th1 and Th17 cells and upregulated C-X-C motif chemokine receptor 1 (CXCR1), CXCR2, IL-17A, IL-22, and IL-23 [146]. In another study, Sobhani et al*.* indicated that the transplantation of fresh feces from CRC patients to germ-free mice could induce hypermethylation of several genes, similar to alteration patterns of CRC patients [147]. A clinical trial of FMT is recruiting for the treatment of metastatic CRC in non-responders to anti-PD-1 therapy (NCT04729322).

## Conclusions

Although there are some review studies on gut microbiota modulation in CRC conditions, there is hardly any review that comprehensively works on the effects of gut microbiota on the efficiency and outcome of the therapeutic strategies. Herein, we tried to have a mechanistic overview of how gut microbiota modulation leads to CRC initiation and progression. Since gut dysbiosis commonly occurs in CRC carcinogenesis, various therapeutic strategies have been developed to alter the gut microbiota, including probiotics, prebiotics, postbiotics, antibiotics, and FMT. These strategies manage CRC treatment via different mechanisms, such as immunomodulation functions, maintaining gut barrier integrity, restoring gut dysbiosis, tumoricidal activities, colonization resistance, and producing anti-cancer products. Despite promising results, there are some concerns related to strategies applied for gut microbiota modulation. For instance, the presence of opportunistic pathogens or virulence factors and the spread of genes responsible for resistance in gut microbial populations are safety concerns of probiotic strategy. Furthermore, side effects, including abdominal pain, mild fever, diarrhea, flatulence, exhaustion, and fatigue, are the main challenges of FMT. Thus, an assessment of the risk–benefit potentials of each strategy in long-term trials and with a large sample should be included in studies to achieve reliable and comprehensive results.

## Data Availability

Not applicable.

## Abbreviations

CRC: Colorectal cancer
GI: Gastrointestinal
ROS: Reactive oxygen species
VEGFR: Vascular endothelial growth factor receptor
BFT: *B. fragilis* Toxin
TLR: Toll-like receptor
CTX: Cyclophosphamide
NO: Nitric oxide
DC: Dendritic cell
IBD: Inflammatory bowel disease
TJ: Tight junction
SCFAs: Short-chain fatty acids
FMT: Fecal microbiota transplantation
CDI: *C. difficile* Infection
